# Corticosterone activity during early weaning reprograms molecular markers in rat gastric secretory cells

**DOI:** 10.1038/srep45867

**Published:** 2017-03-31

**Authors:** Juliana Guimarães Zulian, Larissa Yukari Massarenti Hosoya, Priscila Moreira Figueiredo, Daniela Ogias, Luciana Harumi Osaki, Patricia Gama

**Affiliations:** 1 Department of Cell and Developmental Biology, Institute of Biomedical Sciences, University of Sao Paulo, Sao Paulo, SP, Brazil

## Abstract

Gastric epithelial cells differentiate throughout the third postnatal week in rats, and become completely functional by weaning time. When suckling is interrupted by early weaning (EW), cell proliferation and differentiation change in the gastric mucosa, and regulatory mechanisms might involve corticosterone activity. Here we used EW and RU486 (glucocorticoid receptor antagonist) to investigate the roles of corticosterone on differentiation of mucous neck (MNC) and zymogenic cells (ZC) in rats, and to evaluate whether effects persisted in young adults. MNC give rise to ZC, and mucin 6, Mist1, pepsinogen a5 and pepsinogen C are produced to characterize these cells. We found that in pups, EW augmented the expression of mucins, Mist1 and pepsinogen C at mRNA and protein levels, and it changed the number of MNC and ZC. Corticosterone regulated pepsinogen C expression, and MNC and ZC distributions. Further, the changes on MNC population and pepsinogen C were maintained until early- adult life. Therefore, by using EW as a model for altered corticosterone activity in rats, we demonstrated that the differentiation of secretory epithelial cells is sensitive to the type of nutrient in the lumen. Moreover, this environmental perception activates corticosterone to change maturation and reprogram cellular functions in adulthood.

The adult gastric gland in mammals is organized in isthmus, neck and base regions that are structured by heterogeneous epithelial cell populations. According to the multipotent stem cell prediction^1^, they arise from a niche at isthmus- neck interface^1^,^2^ as pre-mucous surface cells differentiate into mucous surface cells to synthesize mucin 5AC and mucin 1; pre-parietal cells mature to produce HCl, and pre-mucous neck cells give rise to mucous neck cells (MNC). MNC are small, triangular, filled with granules containing glycoproteins (mucin 6 and mucin 1), and localized among parietal cells^3^. Part of this population continuously migrates towards the base of the gland to ultimately differentiate into zymogenic cells (ZC)^1^,^2^,^4^,^5^,^6^. This transition from MNC to ZC depends on the expression of scaling factors that change the secretory apparatus and induce the extension of the apical cytoplasm. During this process, transcription factor Mist1 (codified by *Bhlha15* gene) is essential to coordinate mucous- serous modifications^7^,^8^,^9^,^10^,^11^, and beginning on the third postnatal week, such cells are identified by their morphology under electron microscope and through labeling of glycoproteins, and Mist1^4^,^5^,^6^,^12^. More recently, another proliferative area was described at the base, where stem cells are activated when the gland architecture is disturbed^13^ and the possibility of long-lived, lineage- committed progenitors has been debated^14^.

Mature ZC are localized deeply at base of the gland and produce pepsinogen C (PGC codified by *Pgc* gene). Luminal acid pH converts pepsinogen C into pepsin, the major gastric enzyme^15^. Of note, in suckling rats and mice, ZC also express an immature pepsinogen molecule (*Pga5*) until the third postnatal week^16^,^17^.

Disturbances in the expression of mucins and pepsinogens are part of gastric atrophy and metaplasia processes, which can trigger tumorigenesis in mice and humans^11^. Interestingly, the organization of the gland, as described above, and the digestive functions are only observed and completely effective after suckling to weaning transition, turning it into an important period to analyze growth. Previously, we showed that early weaning (EW) interrupts suckling abruptly, increases gastric epithelial cell proliferation and induces MNC differentiation in rats, and such responses involve EGFR activity through MAPK and Src pathways^18^,^19^. Additionally, EW changes the distribution of TGFβ3 and ghrelin in the gastric mucosa, and collectively they might also contribute to the control of epithelial renewal and maturation during rat growth^20^,^21^.

In rodents, corticosterone is the main glucocorticoid (GC) secreted by adrenal gland, and plasma levels gradually increase from the 14^th^ to the 24^th^ postnatal day, in parallel to suckling- weaning transition^22^. During this period, corticosterone constitutes an important element in the regulation of gastric ontogenesis, and its signaling through glucocorticoid receptor (GR) might interfere with the steps that lead to the differentiation of epithelial cells. Accordingly, treatment with hydrocortisone induces precocious pepsinogen synthesis and activity in developing mice and rats^23^,^24^. Moreover, it is also known that modifications of feeding pattern, such as early weaning, immediately increase corticosterone levels and change GR activity^25^, which might be associated with regulation of gastric growth and accelerated maturation of MNC^18^.

As MNC and ZC differentiate and mature during suckling- weaning transition in rats, we hypothesized that EW would change spatiotemporally the mechanisms involved through the action of corticosterone. In order to test it, we used RU486 (GR antagonist) during early weaning to block corticosterone activity, and studied the expression of genes involved in MNC and ZC differentiation, and evaluated the distribution of molecular markers of these populations. In addition, as the genetic program for growth could have been altered by EW, we also analyzed whether EW and corticosterone effects might be extended to early adult life.

We found that EW increased expression of genes that are part of MNC and ZC differentiation and function, and that molecular and morphological markers followed these responses. Part of such effects is mediated by corticosterone, specifically MNC and ZC distributions as well as pepsinogen C expression, which persisted until early- adult life. We identified as “reprogramming”, the processes that were altered by treatment during the third postnatal week and were maintained to lately in young adults.

Our results contribute significantly to studies that focus on the development of stomach, but also to those directed to investigations connecting cellular functions, nutritional aspects and physiology. Therefore, by taking advantage of the abrupt change induced by early weaning, which alters corticosterone activity, we were able to demonstrate that during gastric gland ontogenesis, the differentiation of secretory epithelial cells is sensitive to the type of nutrient in the lumen. Moreover, this environmental perception activates corticosterone to alter the maturation and functional programs that might remain altered in adulthood.

## Results

### EW and corticosterone effects on body mass gain

The type of nutrient that reaches gastric epithelial cells influences stomach growth during the first three weeks of postnatal development in rats^18^,^19^,^26^,^27^. Early weaning changes the nutritional pattern, and it disrupts different cellular and physiological mechanisms. According to our hypothesis, corticosterone might be effective during EW and accelerate the expression of genes involved in mucous neck cell (MNC) and zymogenic cell (ZC) differentiation. Corticosterone activity was studied after blocking glucocorticoid receptor (GR) with RU486 injection^25^. EW and RU486 treatment were performed on the 15^th^ postnatal day, and so, rats were divided into four groups: suckling control (S), suckling treated with RU486 (SRU), early-weaned control (EW) and early-weaned treated with RU486 (EWRU).

**Figure 1:**
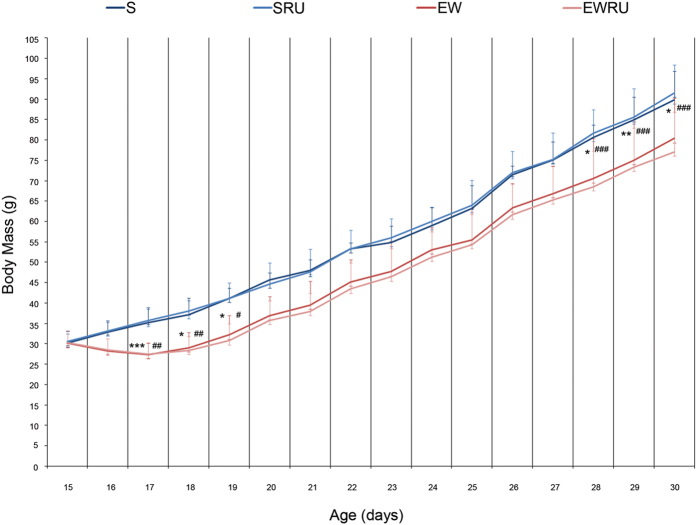
Early weaning and RU486 effects on body mass gain of suckling- control (S), suckling treated with RU486 (SRU), early weaning (EW) and early weaning treated with RU486 (EWRU). Values are means ± SD for each group at each day. Statistics were performed with ANOVA followed by Tukey test for all animals used in the different procedures (n = 7–27 animals/group/age). *P < 0.05; **P < 0.01 and ***P < 00001 versus S group; ^#^*P* < 0.05; ^##^*P* < 0.01 and ^###^*P* < 0.0001 versus SRU group.

Because body mass is a parameter of control for experimental conditions, we firstly monitored it and observed that EW- pups did not gain mass as their suckling counterparts (Fig. 1), as demonstrated by other studies^18^,^20^,^25^. Accordingly, at 17 days, EW rats showed a reduction of body mass that persisted until 19 days. After a period of variation (20 to 27 days), when all animals gained weight similarly, at 30 days, EW animals were smaller than the S group (Fig. 1). Of note, treatment with RU486 did not change body mass.

### Corticosterone functions during EW to change the expression of genes in gastric epithelial maturation

The events involved in the differentiation of MNC and ZC constitute important steps in the maturation and function of gastric gland. The mechanisms by which EW and corticosterone affect them molecularly remain unknown. As GR is predominant in the gastric mucosa when compared to progesterone and mineralocorticoid receptors in gerbils and humans^28^,^29^, and its activity changes with EW in rats^25^, the main target of RU486 treatment was glucocorticoid receptor. We evaluated GR (encoded by *Nr3c1* gene) expression and found that EW and RU486 did not change *Nr3c1* in the gastric mucosa (Supplementary Fig. S1). We also tested the efficiency of RU486 treatment by checking *Sgk1* levels^30^,^31^, and supportive of our aims, we observed that it was immediately (17 days) augmented by EW and reduced by RU486 (Supplementary Fig. 1). Such response was not maintained in 30-day-old rats, indicating that the blockage of corticosterone activity was restricted to the first days after RU486 administration, and any change observed at 30 days would be a long-term effect.

**Figure 2:**
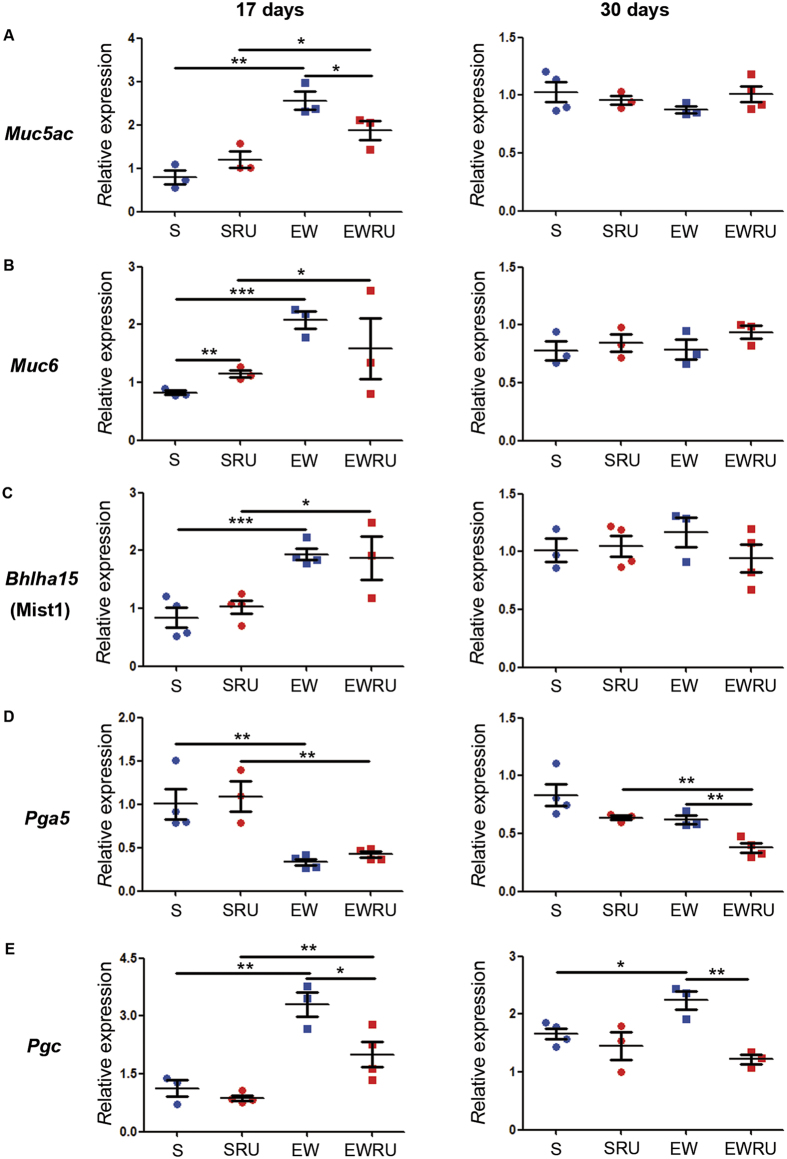
Early weaning and corticosterone change and reprogram gene expression in the gastric mucosa. RT-qPCR was used to detect *Muc5ac* (**A**), *Muc6* (**B**), *Bhlha15* (**C**), *Pga5* (**D**) and *Pgc* (**E**) in suckling (S) or early- weaned (EW) 17 and 30-day- old rats treated or not with RU486. Values shown as means ± S.E.M. (n) = 3–5 animals/group/age. *P < 0.05, **P < 0.01 and ***P < 0.001 after one-tailed Student *t* test performed for dietary condition or RU486 treatment.

Next, we evaluated the genes involved in the differentiation of gastric cells, focusing the secretory ones. We found that at 17 days, EW increased the expression of *Muc5ac* (surface foveolar cell), *Muc6* (MNC), *Bhlha15* (ZC) and *Pgc* (mature pepsinogen in ZC) (Fig. 2). Interestingly, *Pga5* (immature pepsinogen) expression was reduced by EW. Later on (30 days), only *Pgc* remained changed and increased, when S and EW groups were compared (Fig. 2E). This result indicated that the gene that encodes pepsinogen C was reprogrammed by early weaning to a higher rate of transcription in relation to the levels detected in S pups.

By reducing corticosterone effects, we found that in pups, RU486 reduced the expression of *Muc5ac* and *Pgc* when EWRU was compared to EW (Fig. 2A,E). In addition, for these genes, we observed an interaction between corticosterone and dietary condition (S and EW, after two-way ANOVA), which means that hormone effect was dependent on early weaning scenario.

In 30-day-old rats, corticosterone blockage decreased the expression of *Pgc* and *Pga5* in EWRU vs. EW comparison (Fig. 2D,E) and indicated that *Pgc* gene maintained the response described at 17 days. Also for these genes, we detected an interaction between dietary condition and corticosterone treatment (two-way ANOVA).

### Corticosterone induces mucous neck cell differentiation during early weaning

**Figure 3:**
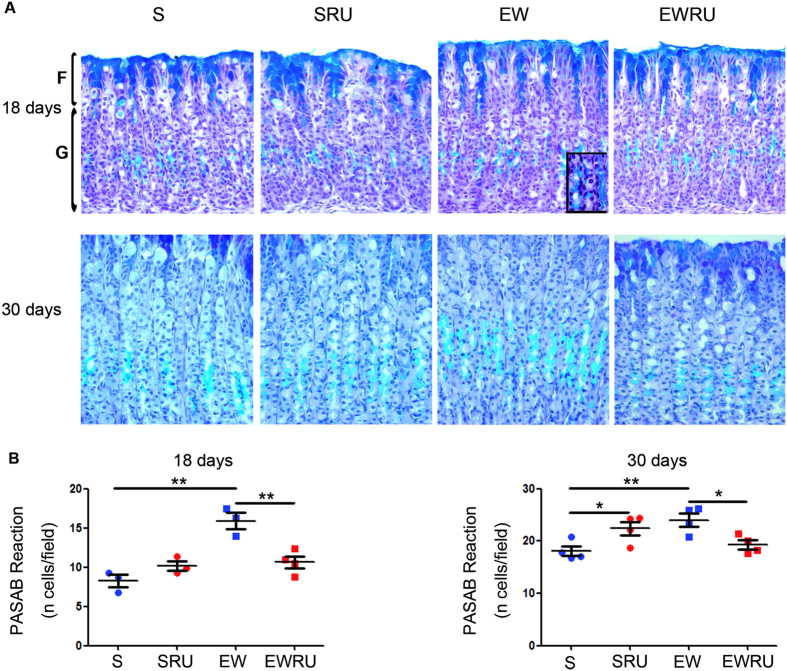
Early weaning and corticosterone reprogram mucous neck cell differentiation. Representative photomicrographs of PASAB histochemistry in the gastric mucosa (*F*- foveola and *G*- gland) in the different groups at 18 and 30 days. Inset shows a detail of MNC stained cytoplasm. Sections were counterstained with Harris hematoxylin. Original magnification: X40 and X100 (inset). The number of mucous neck cells/field (**B**) is represented as means ± S.E.M. (n) = 3–4 for each group. *P < 0.05 and **P < 0.01 after one-tailed Student *t* test for dietary condition or RU486 treatment.

Previously, we showed that as Muc6 transcription increased, MNC population augmented in EW rats^18^. To study the role of corticosterone on MNC differentiation, we used histochemical (PASAB and GSII-FITC) and immunohistochemical (mucin 6) reactions to identify changes in this cell population after blocking GR activity with RU486. Through PASAB technique (Fig. 3A), firstly, we observed that the number of MNC increased when S groups were considered (18- and 30-day- old rats) (Fig. 3B), which is consistent with regular gastric growth. Then, we found that EW augmented mucous neck cell population when compared to S group at 18 days, in agreement with previous results^18^, and such response was maintained at 30 days (Fig. 3B). Importantly, treatment with RU486 induced a reduction in MNC number when EWRU and EW were compared at 18 and 30 days (Fig. 3B). Of note, parietal cells were not focused in the current study, but because of their role in maintenance of gastric mucosa architecture^32^,^33^ we registered their presence along the gland. We detected these cells at 18 and 30 days and experiments are in progress to specifically evaluate them.

**Figure 4:**
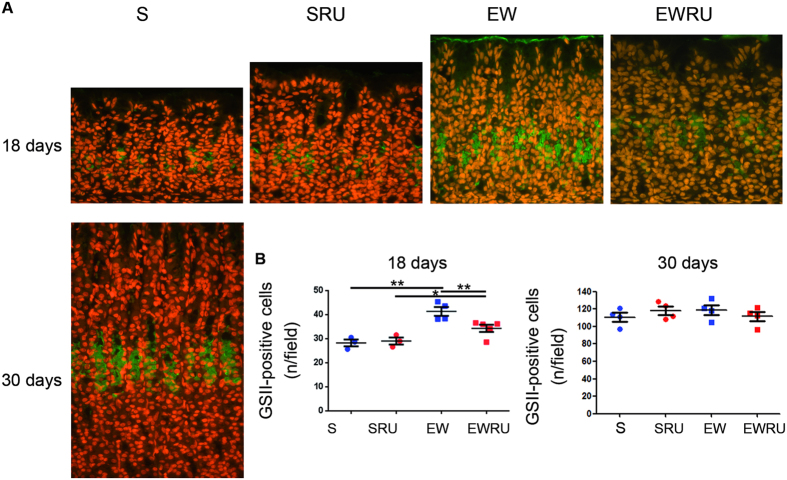
Corticosterone effects on MNC cells differentiation during early weaning. Representative immunofluorescence images of GSII-FITC reaction in the gastric mucosa (**A**) at 18 and 30 days. Original magnification: X40. The number of GSII-FITC positive cells/field (**B**) is represented as means ± S.E.M. (n) = 3–4 for each group. **P < 0.01 after one-tailed Student *t* test for dietary condition or RU486 treatment.

GSII-FITC lectin was also used to detect MNC (Fig. 4A), and we verified that whereas EW increased the number of positive cells, the blockage of corticosterone activity decreased it at 18 days (Fig. 4B). As the population stained by GSII-FITC is larger than that identified by PASAB, mainly due to the inclusion of cells that are in transition to the base, at 30 days, we did not record the same effects (Fig. 4B) described for PASAB. Moreover, we found that the action of corticosterone is dependent on the dietary condition (S or EW), as these parameters were seen to interact (two- way ANOVA).

**Figure 5:**
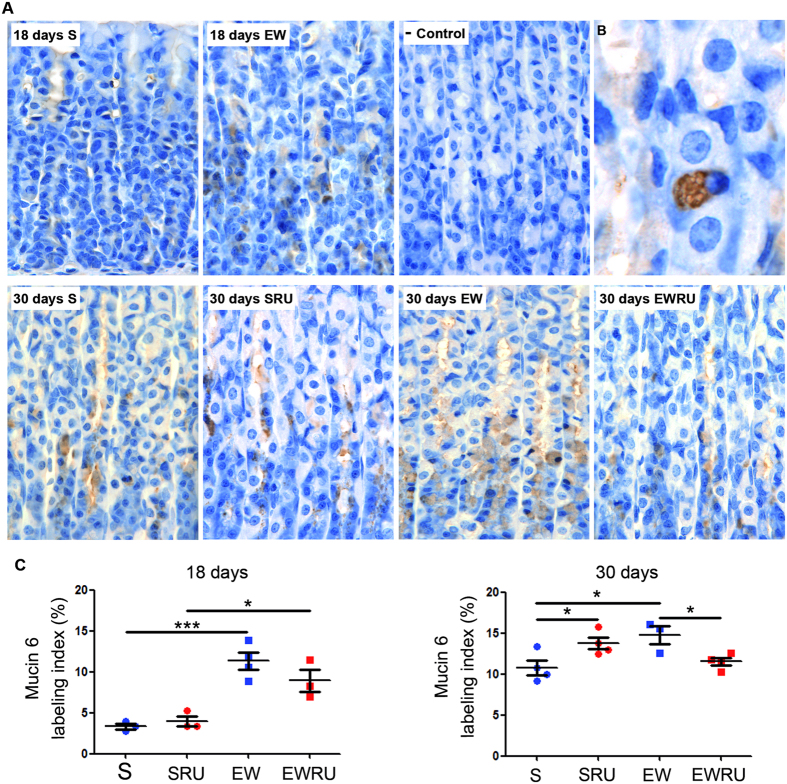
Early weaning and corticosterone change the number of mucin 6- secreting cells. Mucin 6 was scarcely identified in the gastric mucosa of suckling rats (immunohistochemistry developed with DAB+ H_2_O_2_ and counterstained with Mayer’s Hematoxylin), and synthesis increased after EW both in pups and young adults (**A**). Original magnification: X100. Granules containing mucin 6 can be detected in the cytoplasm of mature MNC (**B**) (original magnification at X100 and digital zoom were used). Labeling index (%) is represented as means ± S.E.M. (**C**). (n) = 3–4 for each group. *P < 0.05 and ***P < 0.001 after one-tailed Student *t* test for dietary condition or RU486 treatment.

As gene expression and the glycoprotein content of MNC were affected by corticosterone, next, we used immunolabeling for mucin 6 (Fig. 5A and B). Our analyses demonstrated that EW increased the labeling index for mucin 6 at 18 and 30 days (Fig. 5C), confirming that the abrupt interruption of suckling induced a persistent alteration of this population. Of note, in pups, EW tripled the number of cells when compared to S group (Fig. 5C). Treatment with RU486 reduced the distribution of mucin 6 immunostained cells both in pups and young adults (Fig. 5C).

### MNC transition to ZC: differential roles of EW and corticosterone

**Figure 6:**
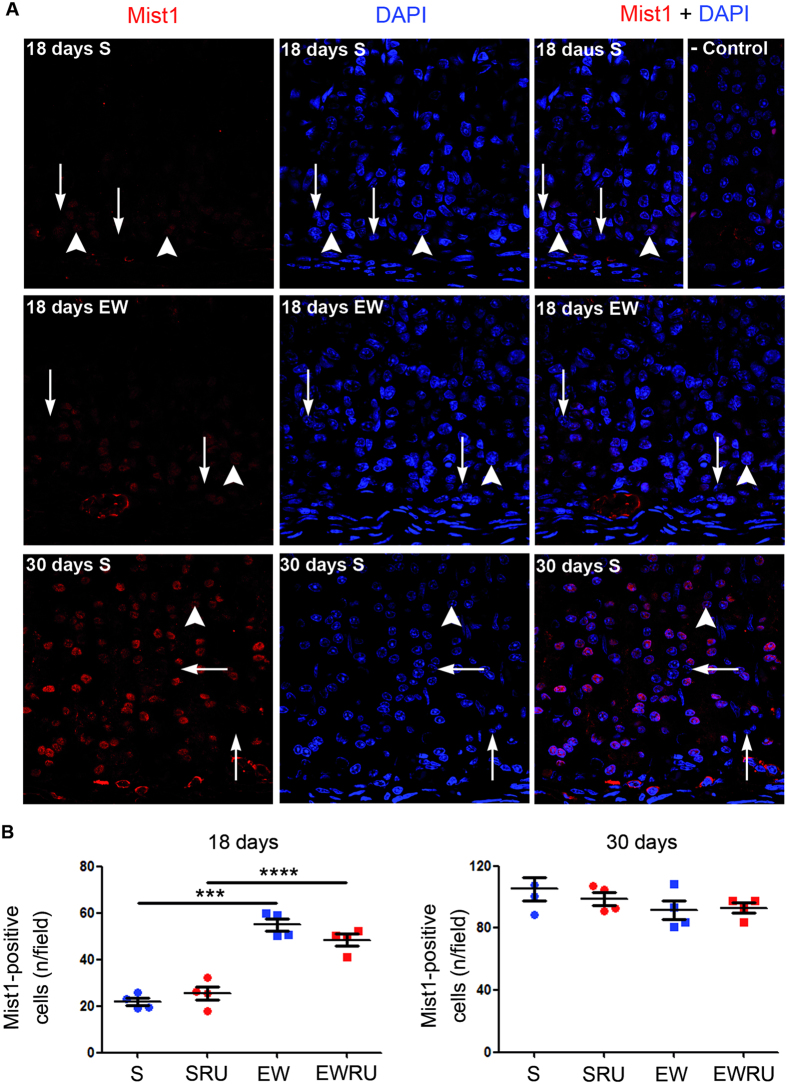
Early weaning increase of Mist1 transcriptional factor in pups does not involve corticosterone action. Mist1 was detected by immunofluorescence (Cy3- conjugated antibody) and labeled nuclei (arrowheads) were seen at the base of the gland (**A**). At 18 days, few Mist1- cells were interspersed among epithelial cells (arrows), but positive population increased with early weaning and aging (30 days). DAPI staining was used to allow gland identification and delimitation. Representative images were obtained under confocal laser scanning microscope using HeNe and Diode lasers at 543 and 505–530 nm of excitation, respectively. Original magnification: X63. (**B**) The number of Mist1-positive cells/field shown as means ± S.E.M. (n) = 3–4 for each group. ***P < 0. 001 and ****P < 0.0001 after one- tailed Student *t* test for dietary condition.

In rodents, gastric glands reach complete maturation during the 3^rd^ postnatal week, concomitant with suckling to weaning transition^18^,^34^. As part of mucous neck cell population differentiates into zymogenic cells^2^,^7^, and the completion of this process depends on Mist1 transcription factor^2^,^10^,^11^, we investigated *Bhlha15* expression, which was up-regulated by EW in pups as described above, and then evaluated the distribution of immunolabeled nuclei. Mist1- positive nuclei were poorly identified in suckling pups, but they increased in number after EW and occupy the whole basal glandular area at 30 days (all groups) (Fig. 6A). Accordingly, Mist1- labeled population augmented three times in S groups from 18 to 30 days (Fig. 6B). We also observed that EW increased the number of Mist1-positive cells when compared to S pups at 18 days (Fig. 6B), and such effect was not recorded at 30 days. In addition, the analyses of EW groups at 18 and 30 days suggested that the major increase in the number of cells/field was detected at 18 days (Fig. 6B). Finally, we observed that RU486 treatment did not alter the distribution of cells immunolabeled for Mist1 (Fig. 6B).

**Figure 7:**
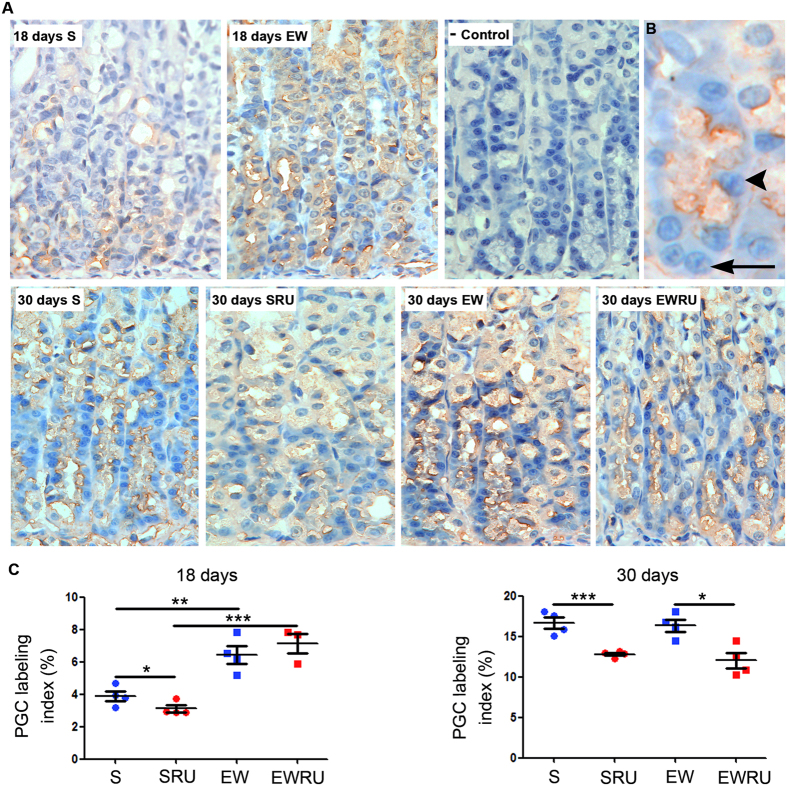
Pepsinogen C- secreting zymogenic cells are reprogrammed by early weaning and corticosterone. Representative photomicrographs from the base of gastric gland show the distribution of immunolabeled cells in the area (**A**). PGC secreting cells (arrowhead) are among epithelial cells (arrow) (**B**). PGC was detected after immunohistochemistry developed with DAB+ H_2_O_2_, counterstained with Mayer’s Hematoxylin. Original magnification: X100 (**A**); digital zoom (**B**). PGC immunolabeling index (%) represented as means ± S.E.M. (**C**). (n) = 3–4 for each group. *P < 0.05; **P < 0.01 and ***P < 0.001 after one tailed Student *t* test to compare dietary condition or RU486 effect.

As mentioned above, zymogenic cells represent a serous-secreting population, and its major product is pepsinogen. Different types and amounts of pepsinogen are produced according to the developmental and functional states of the gastric mucosa^16^,^17^ and, in rats, the PGC marks epithelial maturation^35^. To determine whether corticosterone is involved also in the distribution of these cells, we used immunohistochemistry for pepsinogen and pepsin C (active molecule). Although we were able to evidence PGC-producing ZC at the base of gastric gland, and pepsin C in the lumen and covering parietal cells plasma membrane (Fig. 7A and B), we only considered ZC immunostaining (Fig. 7B). In pups, EW increased PGC immunolabeling index when compared to S group (Fig. 7A,C), and such augment was not altered by RU486 (Fig. 7C). At 30 days, we verified that the response induced by EW was not maintained, although a reduction on the number of immunolabeled ZC was observed after RU486 treatment (Fig. 7A,C).

## Discussion

In adult gastric mucosa, epithelial cell populations arise from a niche at isthmus- neck interface, according to the multipotent stem cell prediction^1^. However, in rats, during the first three weeks of postnatal development, gland areas are not clearly established, and parietal and secretory cells originate from a proliferative compartment that occupies the whole extension of the gland^36^. Importantly, during the second postnatal week (suckling period), mucous neck cells are scarcely seen (Figs 3, 4, 5)^18^ and zymogenic cells produce pepsinogen a5, which is gradually substituted by pepsinogen C^16^,^17^. Once isthmus, neck and base are determined by the end of the third postnatal week (weaning period), mucous neck cells are more numerous and found among parietal cells^3^, which are pivotal for maintenance of the area^32^. Part of MNC population differentiates into ZC^7^,^14^, and such change depends on the activity of hepatocyte nuclear factor 4 α^37^, which controls transcription factor XBP1 (organization of rER)^38^ that directly regulates Mist1 (establishment of apical secretory apparatus)^33^.

**Figure 8:**
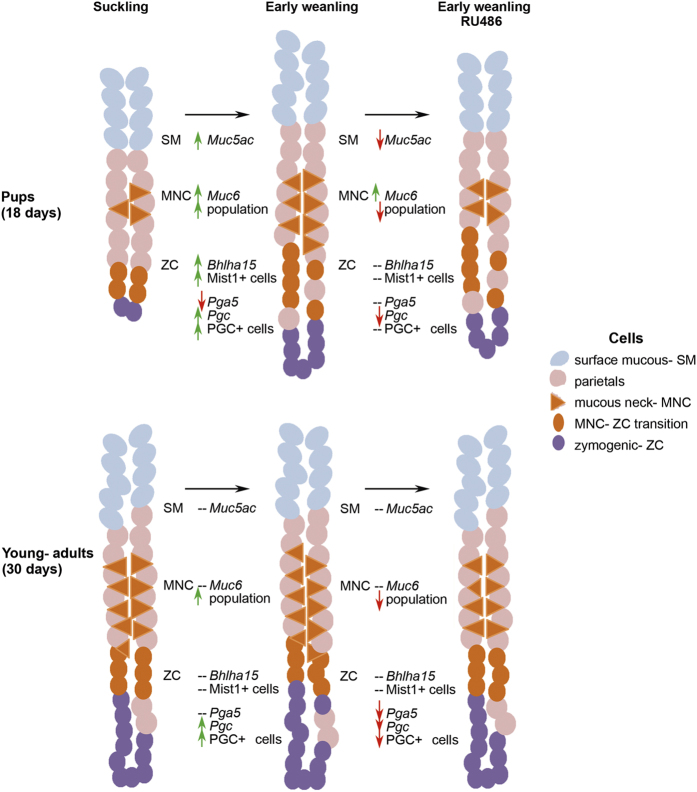
Summary of effects triggered by corticosterone activity during early weaning in rats. Upper and lower panels show schematic gastric gland in pups (18 days) and young- adult rats (30 days), respectively, according to the distribution of cells in suckling and early- weaned rats treated or not with RU486. Effects are noted as increase (⇑), decrease (⇓) and unresponsive (-) for the different markers used to identify genes (italics), proteins and populations of surface mucous (SM), mucous neck (MNC) and zymogenic cells (ZC). Genes, proteins and glycoproteins were studied separately, as indicated, and we demonstrated that corticosterone activity induced the expression of molecular markers involved in the differentiation of secretory cells in pups and part of effects were maintained in young- adults, suggesting a change of program in these cells.

As mentioned above, suckling to weaning transition is critical for gastric development. Consequently, early weaning affects the ontogenesis of stomach^18^,^20^,^27^,^39^, besides changing the maturation of diverse organs and systems in rodents and piglets^40^,^41^. Moreover, as EW induces corticosterone levels and changes receptor (GR) activity^25^, we hypothesized that corticosterone would be involved in the precocious differentiation of gastric secretory cells. Here we reported that part of EW effects is mediated by corticosterone (Fig. 8), and our results demonstrated for the first time some responses were maintained until early- adult life, suggesting that the genetic program, determinant of growth and in course during the first weeks of postnatal development, was altered by the abrupt interruption of suckling.

To study the roles of corticosterone in the maturation of rat stomach, we used RU486, a GR antagonist^42^ that was previously tested and shown to reduce receptor traffic into the nucleus in gastric epithelial cells^25^. We attested RU486 efficiency in our protocol through the analyses of *Sgk1* expression, which is a target of GR in different organs^30^,^43^,^44^. As expected^44^, we found that RU486 did not alter *Nr3c1* (GR) and decreased *Sgk1* mRNA, confirming that this antagonist can be used to reduce corticosterone effect in the gastric mucosa. As both EW and RU486 could influence body weight gain, we also checked it and observed that only dietary change reduced total mass, corroborating other reports^27^,^40^.

Because our aim was to detect the role of corticosterone on differentiation of secretory cells in early- weaned rats, we focused on molecular and morphological markers that allowed the identification of these populations. In the gastric mucosa of corpus region, mucin 5AC is produced only by surface mucous cells associated to Lewis antigens type 1 (Le^a^ and Le^b^), whereas mucin 6 is secreted exclusively in the neck by MNC associated to Lewis antigens type 2 (Le^x^ and Le^y^)^45^. We showed that EW increased *Muc5ac* and *Muc6* expression in pups, and RU486 reversed such response only for *Muc5ac*. Transcription of mucins can be regulated by growth factors^18^,^46^, but synthesis through rER and Golgi Complex involves glycosylation enzymes^47^ that are also controlled by nutritional elements and corticosterone^48^. The actions of GC on mucins are controversial, mainly because of the variability among encoding genes and final glycoproteic products. In nasal epithelial cells, dexamethasone augments the expression of mucins 1, 8 and 16, whereas it decreases mucin 5b^49^,^50^. Interestingly, elevation of mucin 16 is reversed by RU486 in humans^49^, which similarly reduces galactosyl- and α-1,2-fucosyltransferase in the rat intestinal mucosa after hydrocortisone treatment^51^.

Additionally, we used different techniques to identify cells through acid mucous production (alcian blue and GSII- FITC) and mucin 6 synthesis (immunohistochemistry). We demonstrated that EW increased the number of cells labeled for glycoproteins (Figs 3 and 4) and mucin 6 (Fig. 5). RU486 antagonized the response in pups (Figs 3 and 4) and young- adult rats (Figs 3 and 5), indicating that part of EW effects were mediated by corticosterone. Notably, alterations induced by EW and GR blockage in pups persisted to adult age. The maintenance of responses triggered by EW is also described in porcine intestinal barrier, which is injured by the abrupt change of dietary pattern^52^. In the stomach, mucin 6 is important to protect the mucosa from acid pH and *Helicobacter pylori*^53^. Our data showed that EW primed MNC to change the population and its main product (mucin 6), and corticosterone contributed to the regulation of cell density in the mucosa. Moreover, our findings buttress the importance of corticosterone during suckling- weaning transition to the differentiation of mucous neck cells.

Part of MNC differentiates into zymogenic cells^7^,^12^, and the process depends on a cascade of factors, as mentioned above. We currently studied *Bhlha15* (Mist1), *Pga5* and *Pgc* expression and distribution to identify cells that were establishing the apical secretory apparatus for PGC synthesis and secretion^8^,^33^. In EW- pups, we detected increased *Bhlha15* expression, which was not responsive to RU486 (Fig. 2). As during the first three weeks of rat postnatal development, different types and amounts of pepsinogen are secreted by gastric gland^16^, we investigated them, and observed that EW decreased the expression of *Pga5* (immature pepsinogen) and increased *Pgc* (mature pepsinogen) (Fig. 2). In EW young- adults, *Pgc* levels were maintained high, suggesting that the dietary pattern changed and reprogrammed gene expression. The reduction of GR activity (RU486 treatment) decreased *Pgc* expression^23^ and such result also persisted in young- adult rats, which demonstrates GC involvement in ZC differentiation during EW, but also suggests the priming effect of corticosterone on *Pgc*.

In order to identify ZC at the base of the gland, we evaluated Mist1 and PGC distributions and we observed that EW increased the number of immunolabeled cells in pups (Figs 6 and 7), which corroborates previous data^54^. Interestingly, only PGC response seemed to be primed in the gastric mucosa of young- adult rats, as differences in Mist1 were not recorded. We can suggest that although the differentiation of MNC to ZC is influenced by EW, and consequently to nutrient type, corticosterone might function only at pepsinogen regulation.

Therefore, consistent with Barker’s hypothesis about changes of environmental conditions occurring during critical developmental phases and their interference with growth program and functions in adult life^55^,^56^, we demonstrated that early weaning induced modifications in secretory cells in the rat gastric mucosa that persisted to adulthood and part of them depend on corticosterone activity. The potential of these cells to trigger gastric pathologies allied to their sensitivity to dietary and stress conditions point to the need of further studies and policies on suckling to weaning transition and on the effects of increased corticoterone activity during this period.

## Material and Methods

### Animals and early weaning

Wistar rats were mated and pregnant females were housed in isolated cages on the 18^th^ gestational day. Delivery was set as day 0 and litters were culled to 8–9 pups on the 3^rd^ day. Animals were maintained at 22 °C under 12 h light and 12 h dark cycle (lights on at 06h00). All procedures were performed according to the guidelines of National Council of Ethics with Animals (CONCEA) and the protocols were approved by the Ethical Committee for Animal Use from Institute of Biomedical Sciences at University of Sao Paulo (CEUA ICB USP certificates 86/2008; 18/2015). Moreover, experiments were conducted in order to minimize the number of animals and to allow best conditions during treatment.

At 15 days, pups were separated into two groups: suckling (S) and early weaning (EW). The S group was kept with the dam and allowed to suckle freely until regular weaning (21 days) or euthanasia, while EW rats were placed in small plastic cages with water and hydrated powdered chow (Nuvilab CR-1, Quintia SA, Paraná, Brazil) *ad libitum*. Special care was taken with these pups, so that they were offered water and hydrated chow twice a day using disposable Pasteur pipettes. They also received abdominal massage to stimulate the elimination of urine and feces. In order to mimic such handling, S animals were manipulated daily. Rats were euthanized at 17, 18 and 30 days with a 1:1 (v/v) mixture of xylazine and ketamine chloridrates (0.5 ml/100 g body weight) (Anasedan and Dopalen, Vetbrands, Paulínia, SP, Brazil).

### Corticosterone blockage: RU486 (mifepristone) treatment

At 15 days, rats were treated with the GR antagonist RU486 (10 mg/kg body weight) (mifepristone, Sigma-Aldrich, St. Louis, MO, USA) following the procedures previously described^25^. Briefly, pups were injected i.p. with either vehicle (corn oil at 2.5 mg/ml) or RU486 at 17h00 and were divided into four groups: suckling control (S), suckling treated with RU486 (SRU), early-weaned control (EW) and early-weaned treated with RU486 (EWRU).

### RNA isolation, cDNA synthesis and RT-qPCR

At 17 and 30 postnatal days, rat gastric mucosa was scraped and RNA was isolated using TRIzol (Invitrogen, Carlsbad, CA, USA) combined to PureLink^®^ RNA Mini Kit (Invitrogen), following manufacturer’s instruction. Total RNA concentration was determined using NanoDrop (Thermo Fisher Scientific, Waltham, MA USA). Next, 3 μg of total RNA was used to synthesize single-stranded complementary DNAs (cDNAs) with Superscript III Reverse Transcriptase enzyme (200 U/μl, Invitrogen). Quantitative PCR was performed on StepOne Plus Real Time PCR System (Applied Biosystems, Carlsberg, CA, USA). For PCR reactions with Power SYBR^®^ Green PCR Master Mix (Thermo Fisher Scientific), 6 ng of cDNA from each sample were applied and *Gapdh* gene was used as reference. The specificity of primer sequences (listed in Supplementary Table S1) was confirmed via melt-curve analyses. Reactions were run using the following program: 10 min, 95 °C; 15 s, 95 °C, 1 min, 60 °C (40 cycles); 15 s, 95 °C, 1 min 60 °C, 15 s, 95 °C. For PCR reactions with TaqMan^®^ probe assays (Supplementary Table S2) (Thermo Fisher Scientific), 40 ng of cDNA from each rat sample were used and *actb* was taken as reference. Reactions were run following the program: 2 min, 50 °C, 10 min, 95 °C; 15 s, 95 °C, 1 min 60 °C (40 cycles). Data was collected and results were expressed as the relative quantity calculated using 2^−∆∆Ct^ method^57^.

### Histochemical identification and quantification of mucous neck cells

Stomachs were fixed in 10% formaldehyde, embedded in paraffin and 6 μm non- serial sections (intervals of 30 μm) were used to avoid double- counting of cells. After deparaffinization, sections were submitted to histochemical reactions either with Periodic Acid-Schiff/Alcian blue (PAS-AB) or *Griffonia simplicifolia* II lectin conjugated with fluorescein (GSII-FITC, Sigma), following protocols detailed previously^18^. As mucous neck cells are exclusively found in the corpus region of the stomach, we only considered this area in our analyses. For PASAB histochemistry, sections were hydrated, treated with 1% periodic acid (10 min) and stained with Schiff’s reagent (30 min). After washing, AB was applied (45 min). Finally, cell nuclei were counterstained with Harri’s hematoxylin, washed, dehydrated in alcohol series, cleared in xylene and mounted. Samples were analyzed under light microscope (Nikon, Japan) at X800 magnification. The AB-positive cells were counted in the neck of the gland in 10–15 fields per animal and only longitudinally sectioned areas were evaluated.

For GSII- FITC analyses under fluorescence microscopy, sections were treated with 0.3% triton X-100 (room temperature- RT), and then incubated with GSII- FITC lectin (20 μg/ml, 4 h, RT; Vector, Burlingame, CA, USA). Nuclei were counterstained with ethidium bromide (10 μg/ml, 3 min, RT; Invitrogen), following RNase A treatment (5 mg/ml; Calbiochem, San Diego, CA, USA). Five to 10 fields were evaluated per animal at X400 magnification (Axioscope 2, Zeiss, Germany). For both reactions, results were obtained as the number of labeled cells/field per animal. Photomicrographs were acquired using Axioscope 2 and ZEN 2011 (blue edition software, Zeiss).

### Immunohistochemistry for mucin 6, Mist 1 and pepsinogen C, image acquisition and quantification in gastric mucosa

For mucin 6 detection, rat stomachs were fixed in 4% formaldehyde, embedded in paraffin and non-serial 6 μm sections were used as above. After paraffin clearance, sections were hydrated in 0.05 M phosphate buffered saline (PBS) and endogenous peroxidase was inactivated with 3% H_2_O_2_ in methanol (10 min). Antigen retrieval was performed with proteinase K (20 μg/ml, 1 h, RT Gibco, Carlsberg, CA, USA). After washing in PBS, slides were incubated with polyclonal rabbit anti-mucin 6 (8 μg/ml, 4 °C, overnight; cat. number 368623, Santa Cruz Biotechnology, Santa Cruz, CA, USA). After rinsing with PBS, biotin-conjugated secondary antibody was used (5.5 μg/ml, 2 h, RT; cat. number 111-065-003, Jackson ImmunoResearch Laboratories, West Grove, PA, USA), followed by streptavidin-peroxidase complex (8 μg/ml, 2 h, RT; Jackson ImmunoResearch Laboratories). Reaction was developed by H_2_O_2_ in Liquid DAB+ (Dako, Carpinteria, CA, USA), counterstained with 0.1% Mayer’s Hematoxylin, and differentiated in saturated lithium carbonate. For negative control, the primary antibody was omitted. Mucin 6-positive cells were quantified in microscopic fields (8X, Kpl2 Integrative Eye piece with ocular grid, Zeiss) under 100X magnification. Labeled and non-labeled cells were counted in 1 000 epithelial cells and only longitudinally sectioned areas of the neck and base of the gastric glands were used. Results were expressed as the labeling index (%) per rat, which was determined after the number of mucin-6 cells/total of epithelial cells X 100. Photomicrographs were acquired using light microscope (BX51, Olympus, Canada) and Image-Pro^®^ Plus (software version 5.1.2, MediaCybernetics, Silver Spring, MD, USA).

For Mist 1 detection, stomachs were fixed in 10% formaldehyde and non- serial 6 μm sections were treated as above and submitted to antigen retrieval in 0.05 M Tris-HCl (pH 9.0) (water bath in microwave as 5 min at 600 W, and 10 min at 300 W). After washing in PBS, monoclonal mouse anti-mist1 was incubated (8 μg/ml, 4 °C, overnight; cat. number 80984, Santa Cruz Biotechnology). After rinsing in PBS, Cy3- conjugated secondary antibody was incubated (26 μg/ml, 2 h, RT; cat. number 715-165-150, Jackson ImmunoResearch Laboratories). Sections were counterstained with 4′,6-diamidino-2-phenylindole (DAPI; 0.5 μg/ml) and were mounted in Mowiol (Calbiochem). For negative control, the primary antibody was omitted. The nuclei immunolabeled for Mist1 were counted (middle to basal area of gastric glands in corpus region) in 10–15 fields per animal. Only longitudinal sections were considered for evaluation under fluorescence microscope (X 1 000, Axioscope 2). Image acquisition was performed using laser scanning confocal microscope (LSM 780-NLO, ZEN 2011 software, Zeiss) (Centro de Facilidades de Apoio à Pesquisa, CEFAP-ICB/USP).

For pepsinogen C (PGC) detection, the stomach was processed as above for 3 μm non- serial sections. Following the procedures for peroxidase immunostaining, polyclonal goat anti-PGC was incubated (2 μg/ml, 4 °C, overnight; cat. number 51188, Santa Cruz Biotechnology). After washing with PBS, peroxidase-conjugated secondary antibody was used (3.2 μg, 2 h, RT; cat. number 305-035-003, Jackson ImmunoResearch Laboratories) and the reaction was developed and stained as described for mucin 6. PGC-positive cells were quantified and results were expressed as described previously for mucin 6-positive cells.

### Statistical analyses

Differences between two groups were compared by one-tailed unpaired Student *t* test and groups were analyzed by one-way ANOVA, followed by Tukey’s test, as indicated in Results and Figures. The interaction between the effects of the age, diet (S *vs*. EW) and RU486 administration was analyzed by two-way ANOVA. In all analyses differences were considered significant at p < 0.05. Tests were performed using GraphPad Prism 5.03 software (GraphPad Software Inc., La Jolla, CA).

## Additional Information

**How to cite this article:** Guimarães Zulian, J. *et al*. Corticosterone activity during early weaning reprograms molecular markers in rat gastric secretory cells. *Sci. Rep.* **7**, 45867; doi: 10.1038/srep45867 (2017).

**Publisher's note:** Springer Nature remains neutral with regard to jurisdictional claims in published maps and institutional affiliations.

## Supplementary Material

Supplementary Information
