# Supplementary material for: Corticosterone activity during early weaning reprograms molecular markers in rat gastric secretory cells
Source: Sci Rep. 2017 Mar 31;7:45867. doi: 10.1038/srep45867 (PMC5374460; doi:10.1038/srep45867)
Supplement: Supplementary Information [file srep45867-s1.pdf]

Corticosterone activity during early weaning reprograms molecular markers in rat gastric secretory cells

Juliana Guimarães Zulian, Larissa Yukari Massarenti Hosoya, Priscila Moreira Figueiredo, Daniela Ogias, Luciana Harumi Osaki, Patricia Gama*

Department of Cell and Developmental Biology, Institute of Biomedical Sciences, University of Sao Paulo, Sao Paulo, SP, Brazil.

Supplementary Table S1 Primers used for RT-qPCR with Power SYBR® Green PCR Master Mix

Gene/protein	Foward	Reverse	Primer concentration
Gapdh/GAPDH	AGTGCCAGCCTC GTCTCATAG	TAACCAGGCGT CCGATACG	100nM
Sgk1/SGK1	TATGGCCTGCCT CCGTTCT	GTGCCTTGCTGA GTTGGTGAT	150nM
Bhlha15/Mist1	GAACTTGTGCTT GGTCCATCCT	TCCCTATCCTGC GTTCACAAC	150nM

Supplementary Table S2 TaqMan® probe assays used for RT-qPCR

Gene	Assay ID	Codified protein
Actb	Rn00667869_m1	β-actin
Nr3c1	Rn00561369_m1	Glucocorticoids receptor
Muc6	Rn1759814_m1	Mucin 6
Muc5ac	Rn01451270_g1	Mucin 5AC
Pga5	Rn00572739_m1	Pepsinogen 5, group 1
Pgc	Rn00590984_m1	Pepsinogen C

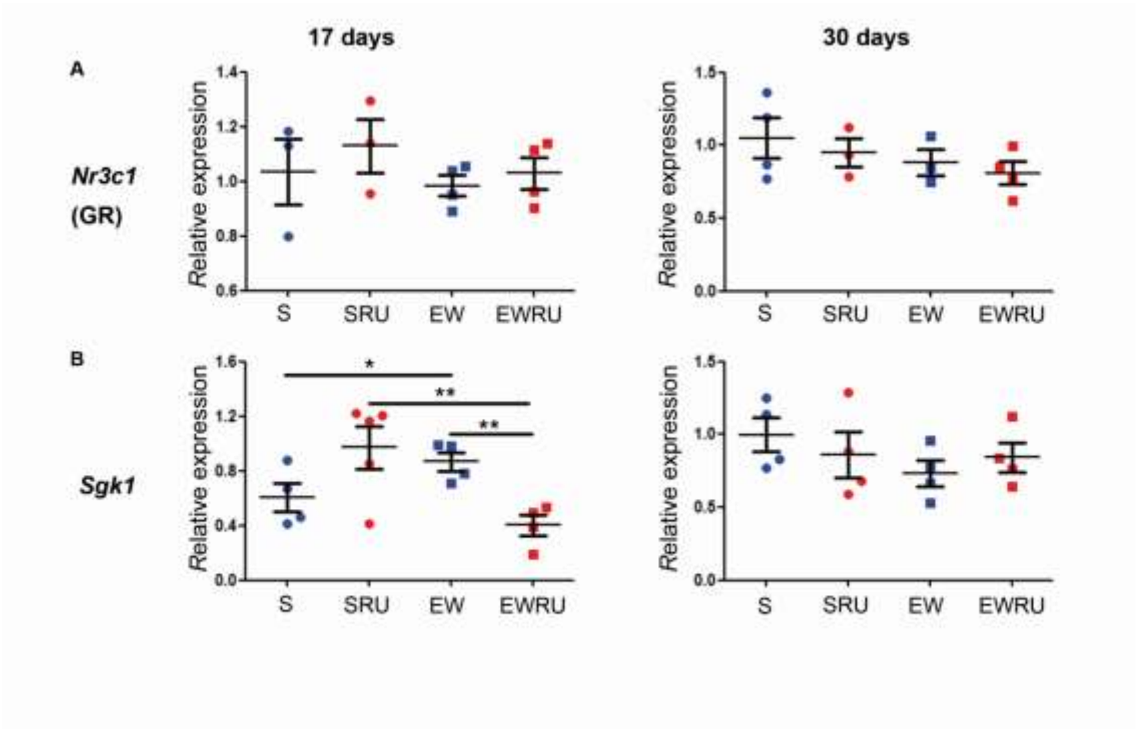

Supplementary Figure 1. *Nr3c1* (GR) and *Sgk1* (SGK1) expression in suckling (S) and early- weaned rats (EW) treated with vehicle (corn oil) or RU486. Values shown as means ± S.E.M. (n) = 3- 5 animals/ group/ age. *P<0.05 and **P<0.01 after one-tailed Student t test to compare dietary condition or RU486 effect.
